# Supplementary material for: Cell-cycle-dependent repression of histone gene transcription by histone H4
Source: Nat Struct Mol Biol. 2026 Jan 5;33(1):145–56. doi: 10.1038/s41594-025-01731-1 (PMC12819152; doi:10.1038/s41594-025-01731-1)
Supplement: Supplementary file 1 — Supplementary Information [file 41594_2025_1731_MOESM1_ESM.pdf]

nature structural & molecular biology

Supplementary information

https://doi.org/10.1038/s41594-025-01731-1

# Cell-cycle-dependent repression of histone gene transcription by histone H4

In the format provided by the authors and unedited

**Supplementary Information**
**Supplementary Table 1. Sample IDs and sequencing results.**
Attached spreadsheet.

**Supplementary Table 2. Histone gene count tables.**
Attached spreadsheet.

**Supplementary Table 3. Differential gene expression between wildtype and *HisCΔ ; 12XWT* wing imaginal discs.**
Attached spreadsheet.

**Bioinformatics for figures**
**For Figure 1:** mapped reads for Mute (BT2579), Mxc (BT3065), Rpb1 (BT542), RNAPII-S5p (BT1781), and RNAPII-S2p (BT3067) were converted to bigwigs with bedtools/genome coverage and displayed in the UCSC genome browser.

**For Figure 2: (a)** .bam files were counted using subReads/featureCounts with option "-o" across a list of all annotated UCSC knownGenes in the dm6 genome assembly. Reads for each histone subtype were summed for each genotype, and counts in each histone subtype in the 12X line were divided by counts in the wildtype line. **(b)** Mapped reads for Mxc (BT3065), Mute (BT3064), H3K4me1 (BT3072), H3K4me2 (BT3068), H3K4me3 (BT3074), H3K27ac (BT3069), H3K36me3 (BT826), H3K9me1 (BT1777), H3K9me2 (BT1778), H3K9me3 (BT1175), H3K27me3 (BT1173), uH2A (BT827), and IgG (BT828) were converted to bigwigs with bedtools/genome coverage and displayed in the UCSC genome browser.

**For Figure 5i:** Mapped reads for Mapped reads for H3K27ac (BT3505), RNAPII-S5p (BT3417), Mxc (BT3195), and histone H4 (BT3510) were converted to bigwigs with bedtools/genome coverage and displayed in the UCSC genome browser.

**For Figure 8: (b,c)** K562 tracks Mapped reads for H3K27ac (BT3514), RNAPII-S5p (SH K5xlin_PolS5P_0320), NPAT (SH NPAT_0217_pool), and histone H4 (BT3518) were converted to bigwigs with bedtools/genome coverage and displayed in the UCSC genome browser. **(d)** Gene scores were counted from .bam files for K562 cell profiling using subReads/featureCounts with option "-o" across a list of all annotated genes in the hg19 (February 2009) genome assembly from -200 bp of annotated gene starts to gene ends. Histone genes are displayed shaded from maximum to minimum counts for each epitope.
