# Supplementary material for: Cell-cycle-dependent repression of histone gene transcription by histone H4
Source: Nat Struct Mol Biol. 2026 Jan 5;33(1):145–56. doi: 10.1038/s41594-025-01731-1 (PMC12819152; doi:10.1038/s41594-025-01731-1)
Supplement: Supplementary file 2 — Reporting Summary [file 41594_2025_1731_MOESM2_ESM.pdf]

nature portfolio

Corresponding author(s): S. Henikoff

Last updated by author(s): Sep 21, 2025

Reporting Summary

Nature Portfolio wishes to improve the reproducibility of the work that we publish. This form provides structure for consistency and transparency in reporting. For further information on Nature Portfolio policies, see our Editorial Policies and the Editorial Policy Checklist.

Statistics

For all statistical analyses, confirm that the following items are present in the figure legend, table legend, main text, or Methods section.

n/a	Confirmed	
☐	☒	The exact sample size (*n*) for each experimental group/condition, given as a discrete number and unit of measurement
☒	☐	A statement on whether measurements were taken from distinct samples or whether the same sample was measured repeatedly
☒	☐	The statistical test(s) used AND whether they are one- or two-sided<br>*Only common tests should be described solely by name; describe more complex techniques in the Methods section.*
☒	☐	A description of all covariates tested
☒	☐	A description of any assumptions or corrections, such as tests of normality and adjustment for multiple comparisons
☒	☐	A full description of the statistical parameters including central tendency (e.g. means) or other basic estimates (e.g. regression coefficient) AND variation (e.g. standard deviation) or associated estimates of uncertainty (e.g. confidence intervals)
☒	☐	For null hypothesis testing, the test statistic (e.g. *F*, *t*, *r*) with confidence intervals, effect sizes, degrees of freedom and *P* value noted<br>*Give P values as exact values whenever suitable.*
☒	☐	For Bayesian analysis, information on the choice of priors and Markov chain Monte Carlo settings
☒	☐	For hierarchical and complex designs, identification of the appropriate level for tests and full reporting of outcomes
☒	☐	Estimates of effect sizes (e.g. Cohen's *d*, Pearson's *r*), indicating how they were calculated

Our web collection on statistics for biologists contains articles on many of the points above.

Software and code

Policy information about availability of computer code

Data collection	The size distributions and molar concentration of libraries were determined using an Agilent 4200 TapeStation. Barcoded CUT&RUN libraries were pooled at approximately equimolar concentration for sequencing. Paired-end 50×50 bp sequencing on the Illumina NextSeq 2000 platform was performed by the Fred Hutchinson Cancer Center Genomics Shared Resources. This yielded 1-40 million reads per antibody. To remove adapter sequences, we preprocessed the reads using cutadapt version 2.9 with parameters -j 8 --nextseq-trim 20 -m 20 -a AGATCGGAAGAGCACACGTCTGAACTCCAGTCA -A AGATCGGAAGAGCGTCGTGTAGGGAAAGAGTGT -Z. Paired-end reads were then aligned to the UCSC hg38 human genome build using Bowtie2 version 2.4.4 with parameters --very-sensitive-local --soft-clipped-unmapped-tlen --dovetail --no-mixed --no-discordant -q --phred33 -I 10 -X 1000.
Data analysis	Bedtools, featureCounts

For manuscripts utilizing custom algorithms or software that are central to the research but not yet described in published literature, software must be made available to editors and reviewers. We strongly encourage code deposition in a community repository (e.g. GitHub). See the Nature Portfolio guidelines for submitting code & software for further information.

Data

Policy information about availability of data

All manuscripts must include a data availability statement. This statement should provide the following information, where applicable:

- Accession codes, unique identifiers, or web links for publicly available datasets
- A description of any restrictions on data availability
- For clinical datasets or third party data, please ensure that the statement adheres to our policy

All sequencing data have been deposited as bigwig files in Gene Expression Omnibus under the accession code GSE280833.

Research involving human participants, their data, or biological material

Policy information about studies with human participants or human data. See also policy information about sex, gender (identity/presentation), and sexual orientation and race, ethnicity and racism.

Reporting on sex and gender	n/a
Reporting on race, ethnicity, or other socially relevant groupings	n/a
Population characteristics	n/a
Recruitment	n/a
Ethics oversight	n/a

Note that full information on the approval of the study protocol must also be provided in the manuscript.

Field-specific reporting

Please select the one below that is the best fit for your research. If you are not sure, read the appropriate sections before making your selection.

☒ Life sciences ☐ Behavioural & social sciences ☐ Ecological, evolutionary & environmental sciences

For a reference copy of the document with all sections, see nature.com/documents/nr-reporting-summary-flat.pdf

Life sciences study design

All studies must disclose on these points even when the disclosure is negative.

Sample size	At least 10 examples of each genotype was characterized for each immunostaining experiment.
Data exclusions	No data were excluded
Replication	Experiments were repeated at least 3 times for chromatin profiling, and at least 5 times for immunostaining experiments.
Randomization	n/a. Multiple individuals of each genotype were characterized.
Blinding	n/a. Experiments characterized genotypes.

Reporting for specific materials, systems and methods

We require information from authors about some types of materials, experimental systems and methods used in many studies. Here, indicate whether each material, system or method listed is relevant to your study. If you are not sure if a list item applies to your research, read the appropriate section before selecting a response.

Materials & experimental systems

n/a	Involved in the study
☐	☒ Antibodies
☐	☒ Eukaryotic cell lines
☐	☐ Palaeontology and archaeology
☐	☒ Animals and other organisms
☐	☐ Clinical data
☐	☐ Dual use research of concern
☐	☐ Plants

Methods

n/a	Involved in the study
☐	☒ ChIP-seq
☐	☐ Flow cytometry
☐	☐ MRI-based neuroimaging

Antibodies

Antibodies used

anti-RNAPII (mouse) Covance Research Products MMS-126R
anti-RNAPII-S5p (rabbit) Cell Signalling Technology D9N5I
anti-RNAPII-S2p (rabbit) Cell Signalling Technology E1Z3G
anti-Mxc (guinea pig) RJ Duronio Mxc-C-1
anti-Mute (guinea pig) RJ Duronio Mute
anti-H3K9me1 (rabbit) Epicypher 13-0029
anti-H3K9me2 (mouse) EMD Millipore 05-1249
anti-H3K9me3 (rabbit) Abcam ab8898
anti-H3K27me3 (rabbit) Cell Signalling Technology C36B11
anti-uH2A (rabbit) Cell Signalling Technology D27C4
anti-H3K4me1 (rabbit) Epicypher 13-0057
anti-H3K4me2 (rabbit) Epicypher 13-0027
anti-H3K4me3 (rabbit) Epicypher 13-0060
anti-H3K27ac (rabbit) Epicypher 13-0059
anti-H3K36me3 (rabbit) Epicypher 13-0058
anti-MPM2 (mouse) DAKO M3514
anti-GFP (rabbit) Cell Signalling Technology D5.1
anti-GFP (mouse) Thermo Fisher Scientific 3E6
anti-rabbit IgG (guinea pig) Antibodies Online ABIN101961
anti-mouse IgG (rabbit) Abcam ab46540
anti-guinea pig IgG (rabbit) Thermo Fisher Scientific PA1-28549
anti-rabbit-FITC (goat) Jackson ImmunoResearch 111-095-144
anti-mouse-FITC (goat) Jackson ImmunoResearch 115-095-166
anti-mouse-RRX (goat) Jackson ImmunoResearch 115-295-166
anti-mouse-Cy5 (goat) Jackson ImmunoResearch 115-175-166
anti-guinea pig-TRITC (goat) Jackson ImmunoResearch 706-025-148
anti-guinea pig-Cy5 (donkey) Jackson ImmunoResearch 706-175-148
anti-histone H4 (mouse) Abcam ab31830
anti-histone H3-S10p (mouse) Abcam Ab14955
anti-NPAT (rabbit) Thermo Fisher Scientific PA565419

Validation

All commercial antibodies were validated by the suppliers. Anti-Mute and anti-Mxc antibodies were validated by R. Duronio (UNC)

Eukaryotic cell lines

Policy information about cell lines and Sex and Gender in Research

Cell line source(s)

Kc167, Drosophila melanogaster female. K562, human female

Authentication

K562 cells were authenticated by ATCC by STR analysis.

Mycoplasma contamination

all cell lines tested negative for mycoplasma.

Commonly misidentified lines
(See ICLAC register)

n/a

Palaeontology and Archaeology

Specimen provenance

Provide provenance information for specimens and describe permits that were obtained for the work (including the name of the issuing authority, the date of issue, and any identifying information). Permits should encompass collection and, where applicable, export.

Specimen deposition

Indicate where the specimens have been deposited to permit free access by other researchers.

Dating methods | *If new dates are provided, describe how they were obtained (e.g. collection, storage, sample pretreatment and measurement), where they were obtained (i.e. lab name), the calibration program and the protocol for quality assurance OR state that no new dates are provided.*

☐ Tick this box to confirm that the raw and calibrated dates are available in the paper or in Supplementary Information.

Ethics oversight | *Identify the organization(s) that approved or provided guidance on the study protocol, OR state that no ethical approval or guidance was required and explain why not.*

Note that full information on the approval of the study protocol must also be provided in the manuscript.

Animals and other research organisms

Policy information about studies involving animals; ARRIVE guidelines recommended for reporting animal research, and Sex and Gender in Research

Laboratory animals	Drosophila melanogaster
Wild animals	The study did not use wild animals.
Reporting on sex	Findings were observed in both male and female cells.
Field-collected samples	The study did not involve animals collected in the field.
Ethics oversight	No oversight was needed for work with Drosophila melanogaster or with the cell lines used.

Note that full information on the approval of the study protocol must also be provided in the manuscript.

Clinical data

Policy information about clinical studies

All manuscripts should comply with the ICMJE guidelines for publication of clinical research and a completed CONSORT checklist must be included with all submissions.

Clinical trial registration	*Provide the trial registration number from ClinicalTrials.gov or an equivalent agency.*
Study protocol	*Note where the full trial protocol can be accessed OR if not available, explain why.*
Data collection	*Describe the settings and locales of data collection, noting the time periods of recruitment and data collection.*
Outcomes	*Describe how you pre-defined primary and secondary outcome measures and how you assessed these measures.*

Dual use research of concern

Policy information about dual use research of concern

Hazards

Could the accidental, deliberate or reckless misuse of agents or technologies generated in the work, or the application of information presented in the manuscript, pose a threat to:

No	Yes	
☐	☐	Public health
☐	☐	National security
☐	☐	Crops and/or livestock
☐	☐	Ecosystems
☐	☐	Any other significant area

Experiments of concern

Does the work involve any of these experiments of concern:

No	Yes	
☐	☐	Demonstrate how to render a vaccine ineffective
☐	☐	Confer resistance to therapeutically useful antibiotics or antiviral agents
☐	☐	Enhance the virulence of a pathogen or render a nonpathogen virulent
☐	☐	Increase transmissibility of a pathogen
☐	☐	Alter the host range of a pathogen
☐	☐	Enable evasion of diagnostic/detection modalities
☐	☐	Enable the weaponization of a biological agent or toxin
☐	☐	Any other potentially harmful combination of experiments and agents

Plants

Seed stocks

Report on the source of all seed stocks or other plant material used. If applicable, state the seed stock centre and catalogue number. If plant specimens were collected from the field, describe the collection location, date and sampling procedures.

Novel plant genotypes

Describe the methods by which all novel plant genotypes were produced. This includes those generated by transgenic approaches, gene editing, chemical/radiation-based mutagenesis and hybridization. For transgenic lines, describe the transformation method, the number of independent lines analyzed and the generation upon which experiments were performed. For gene-edited lines, describe the editor used, the endogenous sequence targeted for editing, the targeting guide RNA sequence (if applicable) and how the editor was applied.

Authentication

Describe any authentication procedures for each seed stock used or novel genotype generated. Describe any experiments used to assess the effect of a mutation and, where applicable, how potential secondary effects (e.g. second site T-DNA insertions, mosiacism, off-target gene editing) were examined.

ChIP-seq

Data deposition

☒ Confirm that both raw and final processed data have been deposited in a public database such as GEO.

☐ Confirm that you have deposited or provided access to graph files (e.g. BED files) for the called peaks.

Data access links
May remain private before publication.

Sequencing data is available for review under the GEO accession GSE280833 at www.ncbi.nlm.nih.gov/geo/query/acc.cgi?acc=GSE280833, enter token wnafcsoilhwxpuz in the box.

Files in database submission

GSM8606309 w_RNAPII_(220119_BT_Dm_BT542_P222)
GSM8606310 w_H3K36ne3_R1_(220406_BT_Hs_BT826_H4)
GSM8606311 w_uH2A_R1_(220406_BT_Hs_BT827_H106)
GSM8606312 w_H3K27me3_R1_(220819_BT_Dm_BT1173_H128)
GSM8606313 w_H3K9me3_R1_(220819_BT_Dm_BT1175_H28)
GSM8606314 w_H3K9me1_R1_(230404_BT_Dm_BT1777_H126)
GSM8606315 w_H3K9me2_R1_(230404_BT_Dm_BT1778_H1)
GSM8606316 w_RNAPII-S5p_R1_(230404_BT_Dm_BT1781_P51)
GSM8606317 12XWT_RNAPII-S5p_R1_(230522_BT_Dm_BT1933_P51)
GSM8606318 12XWT_H3K9me2_R1_(230522_BT_Dm_BT1936_H1)
GSM8606319 12XWT_H3K27me3_R1_(230522_BT_Dm_BT1939_H128)
GSM8606320 12XWT_H3K27ac_R1_(230522_BT_Dm_BT1940_217)
GSM8606321 12XWT_H3K36me3_R1_(230522_BT_Dm_BT1942_283)
GSM8606322 12XWT_uH2A_R1_(230522_BT_Dm_BT1943_H106)
GSM8606323 12XWT_Mute_R1_(230522_BT_Dm_BT1945_mute)
GSM8606324 12XWT_H3K9me2_R2_(230628_BT_Dm_BT1965_H1)
GSM8606325 12XWT_RNAPII-S5p_R2_(230704_BT_Dm_BT1993_P51)
GSM8606326 12XWT_H3K9me2_R3_(230704_BT_Dm_BT1994_H1)
GSM8606327 12XWT_uH2A_R2_(230704_BT_Dm_BT1996_H106)
GSM8606328 12XWT_H3K27ac_R2_(230704_BT_Dm_BT1997_217)
GSM8606329 12XWT_Mxc_R1_(230704_BT_Dm_BT1998_268)
GSM8606330 12XWT_Mxc_R2_(230704_BT_Dm_BT1999_269)
GSM8606331 12XWT_Mute_R2_(230704_BT_Dm_BT2000_mute)
GSM8606332 w_Mute_R1_(240222_BT_Dm_2579_mute)
GSM8606333 12XWT_Mute_R3_(240525_BT_Dm_3052)
GSM8606334 12XWT_Mxc_R3_(240525_BT_Dm_3053)
GSM8606335 12XWT_RNAPII-S2p_R1_(240525_BT_Dm_3055)
GSM8606336 12XWT_RNAPII-S2p_R2_(240525_BT_Dm_3056)
GSM8606337 12XWT_H3K9me3_R1_(240525_BT_Dm_3057)

GSM8606338 12XWT_H3K9me3_R2_(240525_BT_Dm_3058)
GSM8606339 12XWT_H3K4me1_R1_(240525_BT_Dm_3059)
GSM8606340 12XWT_H3K4me2_R1_(240525_BT_Dm_3060)
GSM8606341 12XWT_H3K4me3_R1_(240525_BT_Dm_3061)
GSM8606342 12XWT_H3K4me3_R2_(240525_BT_Dm_3062)
GSM8606343 12XWT_H3K27ac_R3_(240525_BT_Dm_3063)
GSM8606344 w_Mxc_R1_(240525_BT_Dm_3065)
GSM8606345 w_RNAPII-S2p_R1_(240525_BT_Dm_3067)
GSM8606346 w_H3K4me2_R1_(240525_BT_Dm_3068)
GSM8606347 w_H3K27ac_R1_(240525_BT_Dm_3069)
GSM8606348 w_H3K4me1_R1_(240525_BT_Dm_3072)
GSM8606349 w_H3K4me3_R1_(240525_BT_Dm_3074)
GSM8988491 Kc_Mxc_(240709_BT_Dm_3195)
GSM8988492 Kc_RNAPIIS5p_(240820_BT_Dm_3417)
GSM8988493 Kc_H3K27ac_(241125_BT_Dm_3505)
GSM8988494 Kc_histoneH4_(241125_BT_Dm_3510)
GSM8988495 K562_H3K27ac_R1_(241125_BT_Hs_3512)
GSM8988496 K562_H3K27ac_R2_(241125_BT_Hs_3513)
GSM8988497 K562_H3K27ac_R3_(241125_BT_Hs_3514)
GSM8988498 K562_histoneH4_R1_(241125_BT_Hs_3516)
GSM8988499 K562_histoneH4_R2_(241125_BT_Hs_3517)
GSM8988500 K562_histoneH4_R3_(241125_BT_Hs_3518)

Genome browser session
(e.g. UCSC)

n/a

Methodology

Replicates

A least two replicates were performed for each profile.

Sequencing depth

All sequencing was PE50:
Sample ID species genotype antibody epitope mapped reads
BT542 D.melanogaster w P222 Rpb1 19,594,989
BT826 D.melanogaster w H4 H3K36me3 8,952,249
BT827 D.melanogaster w H106 uH2A 8,067,594
BT1173 D.melanogaster w H128 H3K27me3 5,731,799
BT1175 D.melanogaster w H28 H3K9me3 5,201,319
BT1777 D.melanogaster w H126 H3K9me1 3,095,758
BT1778 D.melanogaster w H1 H3K9me2 1,824,927
BT1781 D.melanogaster w P51 RNAPIIS5p 3,055,368
BT1933 D.melanogaster 12XWT P51 RNAPIIS5p 2,349,892
BT1936 D.melanogaster 12XWT H1 H3K9me2 11,809,042
BT1939 D.melanogaster 12XWT H128 H3K27me3 13,191,375
BT1940 D.melanogaster 12XWT 217 H3K27ac 13,722,592
BT1942 D.melanogaster 12XWT 283 H3K36me3 12,842,116
BT1943 D.melanogaster 12XWT H106 uH2A 12,108,154
BT1945 D.melanogaster 12XWT MUTE Mute 760,306
BT1965 D.melanogaster 12XWT H1 H3K9me2 1,785,978
BT1993 D.melanogaster 12XWT P51 RNAPIIS5p 1,949,469
BT1994 D.melanogaster 12XWT H1 H3K9me2 6,829,907
BT1996 D.melanogaster 12XWT H106 uH2A 7,297,519
BT1997 D.melanogaster 12XWT 217 H3K27ac 8,626,044
BT1998 D.melanogaster 12XWT 268 Mxc 1,384,718
BT1999 D.melanogaster 12XWT 269 Mxc 2,751,886
BT2000 D.melanogaster 12XWT Mute Mute 1,532,936
BT2579 D.melanogaster w Mute Mute 4,918,967
BT3052 D.melanogaster 12XWT Mute Mute 3,360,968
BT3053 D.melanogaster 12XWT 268 Mxc 417,996
BT3055 D.melanogaster 12XWT P72 RNAPIIS2p 1,541,295
BT3056 D.melanogaster 12XWT P72 RNAPIIS2p 3,497,752
BT3057 D.melanogaster 12XWT H28 H3K9me3 4,237,975
BT3058 D.melanogaster 12XWT H28 H3K9me3 2,825,340
BT3059 D.melanogaster 12XWT 281 H3K4me1 4,578,654
BT3060 D.melanogaster 12XWT H14 H3K4me2 4,910,423
BT3061 D.melanogaster 12XWT 289 H3K4me3 2,429,885
BT3062 D.melanogaster 12XWT 289 H3K4me3 5,472,314
BT3063 D.melanogaster 12XWT 290 H3K27ac 6,295,838
BT3065 D.melanogaster w 269 Mxc 991,254
BT3067 D.melanogaster w P72 RNAPIIS2p 3,187,095
BT3068 D.melanogaster w H14 H3K4me2 7,731,962
BT3069 D.melanogaster w 217 H3K27ac 8,449,152
BT3072 D.melanogaster w 281 H3K4me1 5,498,442
BT3074 D.melanogaster w 289 H3K4me3 6,092,071
BT3505 D.melanogaster Kc167 cells 290 H3K27ac 8,935,323
BT3417 D.melanogaster Kc167 cells P51 RNAPIIS5p 4,553,069
BT3195 D.melanogaster Kc167 cells 269 Mxc 7,576,135

BT3510 D.melanogaster Kc167 cells H157 histone H4 6,152,565
BT3512 H. sapiens K562 cells 290 H3K27ac 7,015,990
BT3513 H. sapiens K562 cells 290 H3K27ac 6,030,022
BT3514 H. sapiens K562 cells 290 H3K27ac 9,469,857
BT3516 H. sapiens K562 cells H157 histone H4 5,799,455
BT3517 H. sapiens K562 cells H157 histone H4 6,694,997
BT3518 H. sapiens K562 cells H157 histone H4 5,202,544
SH_Hs_K5xlin_PolS5P_1k_0320.DTmarked H. sapiens K562 cells P51 RNAPIIS5p 7,874,209
SH_Hs_K5xlin_PolS5P_2k_0320.DTmarked H. sapiens K562 cells P51 RNAPIIS5p 6,663,749
SH_Hs_K5xlin_PolS5P_3cy_0320.DTmarked H. sapiens K562 cells P51 RNAPIIS5p 4,355,749
SH_Hs_K5xlin_PolS5P_5k_0320.DTmarked H. sapiens K562 cells P51 RNAPIIS5p 6,925,587
SH_Hs_K5xlin_PolS5P_6cy_0320.DTmarked H. sapiens K562 cells P51 RNAPIIS5p 7,329,410
SH_Hs_K5xlin_PolS5P_9cy_0320.DTmarked H. sapiens K562 cells P51 RNAPIIS5p 10,037,905
SH_Hs_K5xlin_PolS5P_10k_0320.DTmarked H. sapiens K562 cells P51 RNAPIIS5p 8,990,835
SH_Hs_K5xlin_PolS5P_12cy_0320.DTmarked H. sapiens K562 cells P51 RNAPIIS5p 8,376,833
SH_Hs_K5xlin_PolS5P_20k_0320.DTmarked H. sapiens K562 cells P51 RNAPIIS5p 11,086,923
SH_Hs_K5xlin_PolS5P_100_0320.DTmarked H. sapiens K562 cells P51 RNAPIIS5p 3,714,156
SH_Hs_K5xlin_PolS5P_200_0320.DTmarked H. sapiens K562 cells P51 RNAPIIS5p 3,937,377
SH_Hs_K5xlin_PolS5P_500_0320.DTmarked H. sapiens K562 cells P51 RNAPIIS5p 6,632,722
SH_Hs_NPA1_20190217.DTmarked H. sapiens K562 cells P281 NPAT 2,429,157
SH_Hs_NPA2_20190217.DTmarked H. sapiens K562 cells P281 NPAT 2,002,911
SH_Hs_NPA4_20190217.DTmarked H. sapiens K562 cells P281 NPAT 1,840,725
SH_Hs_NPA8_20190217.DTmarked H. sapiens K562 cells P281 NPAT 920,075
SH_Hs_NPB1_20190217.DTmarked H. sapiens K562 cells P281 NPAT 2,648,637
SH_Hs_NPB2_20190217.DTmarked H. sapiens K562 cells P281 NPAT 1,619,203
SH_Hs_NPB4_20190217.DTmarked H. sapiens K562 cells P281 NPAT 1,122,716
SH_Hs_NPB8_20190217.DTmarked H. sapiens K562 cells P281 NPAT 640,055

Antibodies

anti-RNAPII (mouse) Covance Research Products MMS-126R
anti-RNAPII-S5p (rabbit) Cell Signalling Technology D9N5I
anti-RNAPII-S2p (rabbit) Cell Signalling Technology E1Z3G
anti-Mxc (guinea pig) RJ Duronio Mxc-C-1
anti-Mute (guinea pig) RJ Duronio Mute
anti-H3K9me1 (rabbit) Epicypher 13-0029
anti-H3K9me2 (mouse) EMD Millipore 05-1249
anti-H3K9me3 (rabbit) Abcam ab8898
anti-H3K27me3 (rabbit) Cell Signalling Technology C36B11
anti-uH2A (rabbit) Cell Signalling Technology D27C4
anti-H3K4me1 (rabbit) Epicypher 13-0057
anti-H3K4me2 (rabbit) Epicypher 13-0027
anti-H3K4me3 (rabbit) Epicypher 13-0060
anti-H3K27ac (rabbit) Epicypher 13-0059
anti-H3K36me3 (rabbit) Epicypher 13-0058
anti-MPM2 (mouse) DAKO M3514
anti-GFP (rabbit) Cell Signalling Technology D5.1
anti-GFP (mouse) Thermo Fisher Scientific 3E6
anti-rabbit IgG (guinea pig) Antibodies Online ABIN101961
anti-mouse IgG (rabbit) Abcam ab46540
anti-guinea pig IgG (rabbit) Thermo Fisher Scientific PA1-28549
anti-histone H4 (mouse) Abcam ab31830
anti-NPAT (rabbit) Thermo Fisher Scientific PA565419

Peak calling parameters

No peaks were called.

Data quality

Quality was assessed by correlation between replicates.

Software

bamtools, featureCounts

Flow Cytometry

Plots

Confirm that:

☐ The axis labels state the marker and fluorochrome used (e.g. CD4-FITC).

☐ The axis scales are clearly visible. Include numbers along axes only for bottom left plot of group (a 'group' is an analysis of identical markers).

☐ All plots are contour plots with outliers or pseudocolor plots.

☐ A numerical value for number of cells or percentage (with statistics) is provided.

Methodology

Sample preparation	*Describe the sample preparation, detailing the biological source of the cells and any tissue processing steps used.*
Instrument	*Identify the instrument used for data collection, specifying make and model number.*
Software	*Describe the software used to collect and analyze the flow cytometry data. For custom code that has been deposited into a community repository, provide accession details.*
Cell population abundance	*Describe the abundance of the relevant cell populations within post-sort fractions, providing details on the purity of the samples and how it was determined.*
Gating strategy	*Describe the gating strategy used for all relevant experiments, specifying the preliminary FSC/SSC gates of the starting cell population, indicating where boundaries between "positive" and "negative" staining cell populations are defined.*

☐ Tick this box to confirm that a figure exemplifying the gating strategy is provided in the Supplementary Information.

Magnetic resonance imaging

Experimental design

Design type	*Indicate task or resting state; event-related or block design.*
Design specifications	*Specify the number of blocks, trials or experimental units per session and/or subject, and specify the length of each trial or block (if trials are blocked) and interval between trials.*
Behavioral performance measures	*State number and/or type of variables recorded (e.g. correct button press, response time) and what statistics were used to establish that the subjects were performing the task as expected (e.g. mean, range, and/or standard deviation across subjects).*

Acquisition

Imaging type(s)	*Specify: functional, structural, diffusion, perfusion.*
Field strength	*Specify in Tesla*
Sequence & imaging parameters	*Specify the pulse sequence type (gradient echo, spin echo, etc.), imaging type (EPI, spiral, etc.), field of view, matrix size, slice thickness, orientation and TE/TR/flip angle.*
Area of acquisition	*State whether a whole brain scan was used OR define the area of acquisition, describing how the region was determined.*

Diffusion MRI ☐ Used ☐ Not used

Preprocessing

Preprocessing software	*Provide detail on software version and revision number and on specific parameters (model/functions, brain extraction, segmentation, smoothing kernel size, etc.).*
Normalization	*If data were normalized/standardized, describe the approach(es): specify linear or non-linear and define image types used for transformation OR indicate that data were not normalized and explain rationale for lack of normalization.*
Normalization template	*Describe the template used for normalization/transformation, specifying subject space or group standardized space (e.g. original Talairach, MNI305, ICBM152) OR indicate that the data were not normalized.*
Noise and artifact removal	*Describe your procedure(s) for artifact and structured noise removal, specifying motion parameters, tissue signals and physiological signals (heart rate, respiration).*
Volume censoring	*Define your software and/or method and criteria for volume censoring, and state the extent of such censoring.*

Statistical modeling & inference

Model type and settings	*Specify type (mass univariate, multivariate, RSA, predictive, etc.) and describe essential details of the model at the first and second levels (e.g. fixed, random or mixed effects; drift or auto-correlation).*
Effect(s) tested	*Define precise effect in terms of the task or stimulus conditions instead of psychological concepts and indicate whether ANOVA or factorial designs were used.*

Specify type of analysis: ☐ Whole brain ☐ ROI-based ☐ Both

Statistic type for inference

(See Eklund et al. 2016)

Specify voxel-wise or cluster-wise and report all relevant parameters for cluster-wise methods.

Correction

Describe the type of correction and how it is obtained for multiple comparisons (e.g. FWE, FDR, permutation or Monte Carlo).

Models & analysis

n/a	Involved in the study
☐	☐ Functional and/or effective connectivity
☐	☐ Graph analysis
☐	☐ Multivariate modeling or predictive analysis

Functional and/or effective connectivity

Report the measures of dependence used and the model details (e.g. Pearson correlation, partial correlation, mutual information).

Graph analysis

Report the dependent variable and connectivity measure, specifying weighted graph or binarized graph, subject- or group-level, and the global and/or node summaries used (e.g. clustering coefficient, efficiency, etc.).

Multivariate modeling and predictive analysis

Specify independent variables, features extraction and dimension reduction, model, training and evaluation metrics.
